# Tailored Lignin Fractions via Ionic Liquid Pretreatment for Sustainable Polymer Systems

**DOI:** 10.3390/molecules30122630

**Published:** 2025-06-17

**Authors:** Sharib Khan, Daniel Rauber, Udayakumar Veerabagu, Ruijie Wu, Christopher W. M. Kay, Chunlin Xu, Sabarathinam Shanmugam, Timo Kikas

**Affiliations:** 1 Biosystems Engineering, Institute of Forestry and Engineering, Estonian University of Life Sciences, Kreutzwaldi 56, 51006 Tartu, Estonia; sharib.khan@emu.ee (S.K.); udayakumar.veerabagu@emu.ee (U.V.); 2 Department of Chemistry, Saarland University, Campus B2.2, 66123 Saarbrücken, Germany; daniel.rauber@uni-saarland.de (D.R.); christopher.kay@uni-saarland.de (C.W.M.K.); 3 Laboratory of Natural Materials Technology, Åbo Akademi University, Henrikinkatu 2, FI-20500 Turku, Finland; ruijie.wu@abo.fi (R.W.); chunlin.xu@abo.fi (C.X.); 4 London Centre for Nanotechnology, University College London, 17-19 Gordon Street, London WC1H 0AH, UK

**Keywords:** lignin fractionation, protic ionic liquid, biopolymer, membrane, biorefinery

## Abstract

The valorization of advanced biorefinery lignins remains a significant challenge, owing to the presence of residual carbohydrates. These lignin-associated carbohydrates hinder lignin purification, reduce its homogeneity, and complicate chemical modifications, ultimately limiting the efficient conversion of lignin into high-value products such as chemicals and materials. This study presents a protic ionic liquid-based lignin fractionation process developed using softwood biomass. Triethylammonium methane sulfonate ([N222H][OMS]) was used to fractionate *Pinus sylvestris*, yielding two distinct fractions: a low-molecular-weight lignin fraction (LF) and a high-molecular-weight lignin fraction (HF). The extracted fractions were comprehensively characterized using nuclear magnetic resonance (NMR) to quantify changes in interunit linkages (*β-O-4*, *β-5*, and *β-β*) and hydroxyl group distribution, whereas methanolysis gas chromatography/mass spectrometry (GC/MS) was used to quantify residual carbohydrates. The fractionation process achieved LF and HF yields of approximately 70.32% and 17.58%, respectively. Further analysis revealed that the HF contained 59.92 ± 2.12 mg/g carbohydrates, whereas the LF contained only 27.37 ± 1.13 mg/g. These findings underscore the effectiveness of the protic ionic liquid fractionation process in reducing carbohydrate impurities and enhancing lignin purity, paving the way for the more efficient utilization of lignin in value-added applications.

## 1. Introduction

Plant biomass is a key renewable resource for producing sustainable chemicals and materials in the future [1]. Among its major components, lignin constitutes approximately 15–27% of the total biomass and has significant potential as an alternative to petrochemical feedstock [2]. This heterogeneous biopolymer contains many functional groups, including phenolic, carboxylic, carbonyl, aliphatic, and methoxy groups, enabling its use as a precursor in certain high-value applications such as bio-based polymers, chemicals, and materials [3]. Although, these renewable precursors have the desired ability to be substituted in polyurethanes, resins, and coatings, their selective extraction from black liquor remains a significant bottleneck [4]. Traditional pulp and paper industries primarily process lignin black liquor, with kraft lignin exhibiting recalcitrance owing to its structural complexity [5]. In contrast, modern biorefinery lignins, such as hydrolysis lignin originating from sugar-based refinery processes for wood fractionation, offer opportunities to tailor lignin fractionation to distinct molecular weight (Mw) fractions, thereby enhancing the solubility and functionality of high-value lignin applications [6,7]. Despite this potential, biorefineries predominantly prioritize carbohydrate-derived biofuels (e.g., bioethanol), releasing lignin to low-value roles, such as those mostly used for producing energy [3]. The presence of carbohydrate-associated impurities further complicates lignin processing because covalently linked carbohydrates significantly influence their solubility and enhance their recalcitrance [8]. Therefore, lignin and its carbohydrate-linked fractions should be considered key intermediates in lignin valorization. Advancements in pulp mills and biorefinery technologies offer greater flexibility for tuning lignin properties and tailoring them to meet specific requirements for high-value applications [9].

Biorefinery processes, including conventional chemical pretreatments (e.g., alkaline, acid, or organosolv) and chemical-free methods (e.g., steam, nitrogen explosion or mechanical milling), have demonstrated utility in modulating lignin properties for downstream applications [10]. However, these approaches often involve high energy consumption, harsh operating conditions, and environmental concerns, owing to toxic chemical byproducts [11]. In contrast, protic ionic liquid (PIL) pretreatment has emerged as a sustainable and efficient alternative that offers distinct advantages for lignin modification under mild thermal conditions while aligning with green chemistry principles [12]. Recent studies of PILs have highlighted the environmental and technical benefits of traditional biorefinery approaches. For instance, PILs, derived from low-cost amines and organic acids, exhibit high biocompatibility, recyclability, and tunable solvation properties, thereby enabling selective lignin extraction [13,14]. Several studies have highlighted that PILs such as ammonium-based ions achieve a >90% recovery efficiency over multiple cycles, thereby reducing waste generation and operational costs [15,16,17]. Additionally, the low volatility and non-flammability of PILs address safety concerns associated with volatile organic solvents [18]. Thus, PIL pretreatment represents a paradigm shift in sustainable biorefining, offering energy-efficient lignin extraction with superior quality and reduced environmental impacts.

In this study, an integrated PIL pretreatment of softwood biomass with a single-solvent membrane fractionation of lignin was accomplished, leveraging PILs’ hydrogen-bonding capacity of PILs for the selective separation of lignin fractions with controlled Mw distributions and reduced carbohydrate impurities. Selective lignin fractionation from intact lignocellulosic biomass is essential for optimizing fractionation strategies, enabling the substantial production of lignin and carbohydrate-rich lignin that can be substituted in certain high-value applications, such as chemicals, biobased nanocomposites, foams, and fiber-based packaging [19,20]. Therefore, the further investigation of the selective separation of these fractions is critical to unlock their full potential in modern biorefineries [21]. Recently, Balakshin and colleagues emphasized that effective lignin engineering or fractionation requires separation not only by molecular weight, but also by chemical composition. The removal of carbohydrate impurities is a crucial step in integrating lignin into biorefinery processes and enhancing its valorization for targeted applications [3,22,23]. Lignin fractionation methods including multiple-solvent extraction, acid precipitation, and membrane ultrafiltration, primarily depend on the solubilization of lignins in various solvents [24]. These methods typically target high-molecular-weight lignin fractions, to reduce heterogeneity and refine the molecular weight distribution. However, the presence of various carbohydrate impurities poses serious challenges in achieving the effective separation and characterization of lignin [25]. Solvents with strong hydrogen-bonding capacities, such as ethanol, enhance lignin dissolution and can be employed to reduce the polydispersity index (PDI) of lignin fractions through hydrogen-bonding with lignin structures [26,27,28]. Thus, integrating PIL pretreatment with subsequent solvent fractionation and characterization is an effective strategy for obtaining low-heterogeneity lignin fractions, while considering carbohydrate impurities, molar weight distribution, functional hydroxyl content, and aromatic unit composition [29].

Commercial lignin extraction techniques such as the LignoBoost process face challenges, such as colloidal formation, condensation reactions, and low purity during pH-driven precipitation [30]. These limitations complicate filtration and separation processes, ultimately yielding lignin fractions of relatively low purity. In contrast, ultrafiltration fractionation offers greater control over the molar weight via membrane cutoff, coupled with cost-effectiveness and low energy requirements. However, membranes have a lower shelf life and fouling associated with the high molarity of NaOH or other bases [31]. To overcome these barriers, an integrated strategy combining PIL pretreatment and membrane fractionation was developed in this study. Ethanol, which has a strong hydrogen-bonding capacity, was employed to dissolve and fractionate the lignin extracted from the PIL using membrane filtration. This integrated approach not only minimizes lignin heterogeneity but also separates carbohydrate-conjugated lignin. The comprehensive characterization of the extracted low-molecular-weight lignin fraction (LF) and high-molecular-weight lignin fraction (HF) lignins using advanced analytical techniques provides valuable insights into their structural and functional properties. This novel methodology bridges the gap between biorefinery scalability and lignin valorization, offering a circular economic solution for transforming underutilized lignin into tailored bioproducts.

## 2. Results and Discussion

### 2.1. Ionic Liquid Fractionation of Pine Wood Biomass

**Figure 1:**
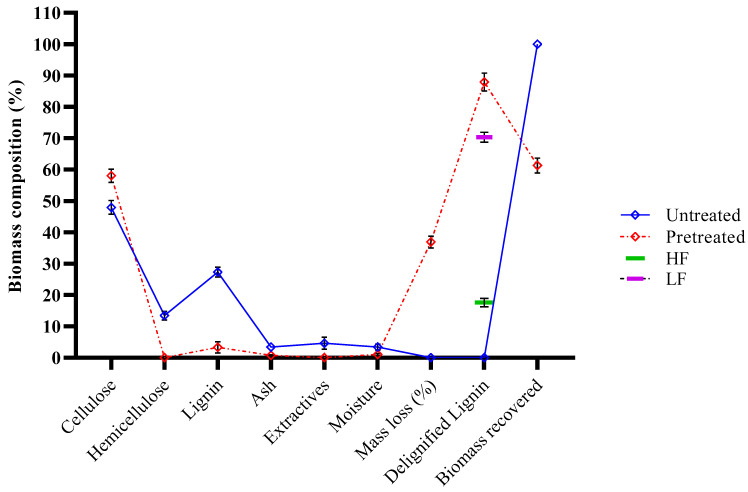
Pretreatment and fractionation of PWB using [N222H][OMS] PIL.

The pretreatment of pine wood biomass (PWB) using a protic ionic liquid (PIL) [N222H][OMS] was investigated, with a focus on maximizing lignin extraction efficiency. The compositional analysis of the treated biomass revealed significant alterations in lignin, cellulose, and hemicellulose contents compared to untreated PWB, as illustrated in Figure 1. The pretreatment with [N222H][OMS] at 180 °C for 90 min with a 1:3 biomass/PIL ratio (*w*/*w*) resulted in over 80% delignification, demonstrating the high efficacy of PIL for lignin removal. This process also led to a substantial reduction in hemicellulose content, likely because of the pretreatment conditions and acidic nature of [N222H][OMS], which effectively disrupted the inter- and intramolecular hydrogen bonds within the PWB [17]. Furthermore, the cellulose content increased slightly after the pretreatment, whereas the residual lignin content in the pulp was significantly reduced. Thus, elaborating on the delignification process exposes the fibers to a larger surface area, thereby enhancing cellulose accessibility [14,18]. After delignification, the ionic liquid lignin, solubilized in ethanol, was fractionated using a pressure filtration system. The yields of the high-molecular-weight lignin fraction (HF) and low-molecular-weight fraction (LF) were 17.58% and 70.32%, respectively. In a related study on lignin fractionation, Liu et al. conducted sequential extractions of industrial softwood kraft lignin using solvents with varying hydrogen-bonding capacities [25]. They observed that carbohydrate signals were prominent in lignin fractions extracted using ethanol, methanol, and dioxane solvents with high hydrogen-bonding abilities. This finding suggests that the solubility of lignin, particularly when covalently linked to carbohydrates, is strongly influenced by the hydrogen-bonding capacity of the solvent. Consequently, selective lignin separation with minimal carbohydrate contamination can be achieved by optimizing solvent conditions. In this study, ethanol was employed to fractionate the ionic liquid lignin, allowing for the analysis of the carbohydrate content across two different fractions.

### 2.2. Differences in the Molecular Weights of HF and LF Fractions

**Figure 2:**
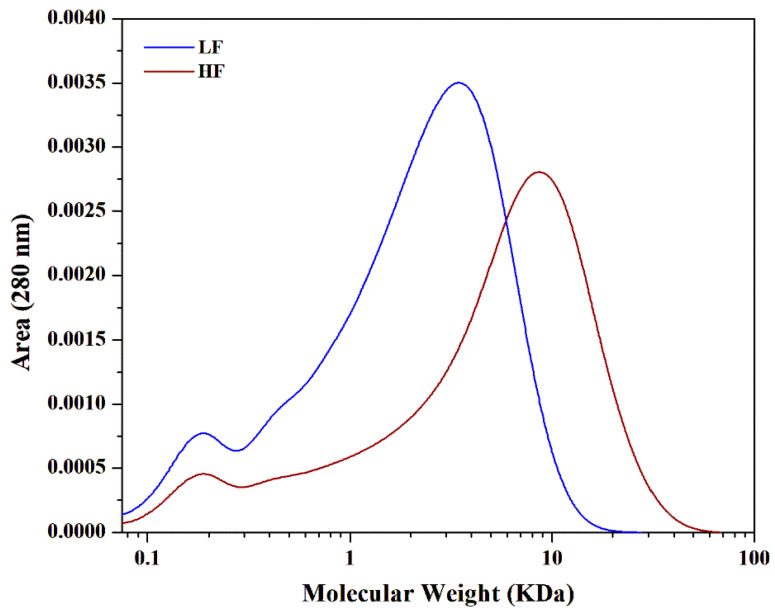
Size Exclusion Chromatogram of higher-molecular-weight lignin fraction (HF) and lower-molecular-weight lignin fraction (LF).

The changes in the weight-averaged molecular weight (Mw) and number-averaged molecular weight (Mn) were measured using Gel permeation chromatography (GPC). The relative molecular weight distribution method, previously established by our group [32], was used to determine the changes in the sizes of different lignin polymers. The Mw values of HF and LF were 9535 and 3342 g mol^−1^, respectively (Table 1). Notably, the molecular weight distribution curve of the HF exhibited low-intensity peaks, whereas that of the LF displayed high-intensity peaks within the 0–30 kDa molecular weight range, as shown in Figure 2. Additionally, variations in the polydispersity index (PDI) of lignins typically reflect the breadth of polymer distribution. In this study, the PDI for the HF was 6.56, compared with 3.97 for the LF, indicating a broader molecular weight distribution in the HF. The results also indicated that some minor HF fractions remained undissolved, whereas LF polymers were fully solubilized. This difference in solubility may be attributed to the lower carbohydrate content of the LF, as previously reported [25,26,29]. Furthermore, the higher solubility of the LF in ethanol after ionic liquid pretreatment (ionoSolv process) could be due to its reduced carbohydrate content. The ionoSolv pretreatment process selectively extracts lignin and hemicellulose, resulting in a cellulose-rich residue [18,33]. This suggests that the HF may contain a higher proportion of carbohydrates, which could provide insights into the structural mechanisms governing sugar regulation during the fractionation and purification of lignins [34]. To fully understand this phenomenon, the further characterization of the lignin fractions is necessary.

### 2.3. Intensities of Different Functional Groups in HF and LF Compared with the Pretreated Pulp

**Figure 3:**
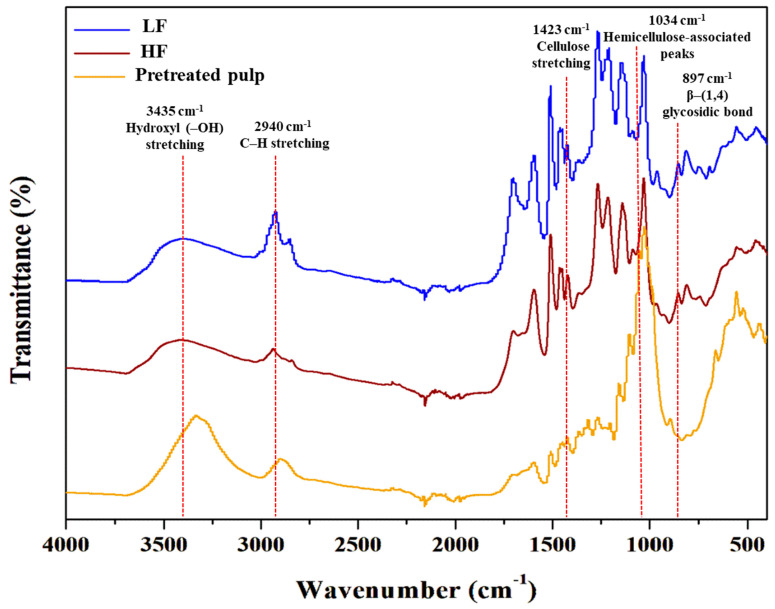
ATR−FTIR infrared spectra of pretreated pulp, HF, and LF.

ATR-FTIR (Attenuated total reflectance/Fourier transform infrared) is versatile analytical technique widely used for identifying functional groups and classes of compounds, making it a valuable tool for validating lignin samples in conjunction with other analytical methods. The ATR-FTIR spectra of the pretreated pulp, HF, and LF revealed distinct changes, as shown in Figure 3, particularly in the lignin fingerprint region (1700–1000 cm^−1^). Specific spectral variations were detected at 2940–2942 cm^−1^, corresponding to alkyl C–H stretching in methyl and methylene groups, and at 910 cm^−1^, indicating out-of-plane aromatic C–H deformations [35]. The purified lignin spectra exhibited characteristic bands corresponding to hydroxyl (–OH) stretching vibrations in both aliphatic and aromatic structures (3435 cm^−1^), C–H bond stretching in methylene and methyl groups (2940 and 2880 cm^−1^), and vibrational modes of the aromatic backbone (1511, 1456 and 1420 cm^−1^) [36]. Additionally, distinct hemicellulose-associated peaks (1034 cm^−1^) and sugar-derived features, including C–O–C stretching at 1160 cm^−1^ (linked to hexose compounds), were identified [37]. Variations in chemical shifts and absorption band intensities at 3435 cm^−1^ may be attributed to differences in hydroxyl group types [38]. The carbohydrate-associated absorption bands, such as those at 896 cm^−1^ (*β*-glycosidic bonds), indicate a relatively higher hemicellulose content in the HF and pretreated pulp [37,39]. Additionally, the pretreated pulp exhibited characteristic peaks corresponding to the crystallinity of cellulose, including the β–(1,4) glycosidic bond (897 cm^−1^) and the ordered cellulose structure (1423 cm^−1^) [40]. These observations align with the established literature on cellulose and lignin characterization using FTIR spectroscopy. This trend suggests the presence of varying amounts of carbohydrate residues in the samples, as lignin with a lower carbohydrate content typically exhibits a higher resolution and intensity in the FTIR spectra. The inclusion of sugars in the samples likely contributed to the observed differences in spectral intensity, as carbohydrates can interfere with the clarity of the lignin-specific peaks. These findings highlight the need for high-resolution analytical techniques to further elucidate the role of carbohydrates in lignin fractions and assess lignin more accurately. Advanced methods, such as 2D NMR or GC/MS, can provide deeper insights into the structural composition and carbohydrate content of these fractions.

### 2.4. Understanding the Peculiarities in HF and LF Using 2D HSQC NMR

^1^H–^13^C Heteronuclear Single Quantum Coherence (HSQC) spectroscopy was employed to investigate the structural changes in lignin following an integrated protic ionic liquid pretreatment and fractionation of softwood biomass. The HSQC spectra were analyzed in two key regions: the aliphatic oxygenated side-chain region (δC/δH 0–100/0.0–5.0) and aromatic/unsaturated region (δC/δH 100–140/5.0–8.0). Peak assignments were made based on comparisons with the previous literature, as detailed in the Supplementary Information (Table S1).

**Figure 4:**
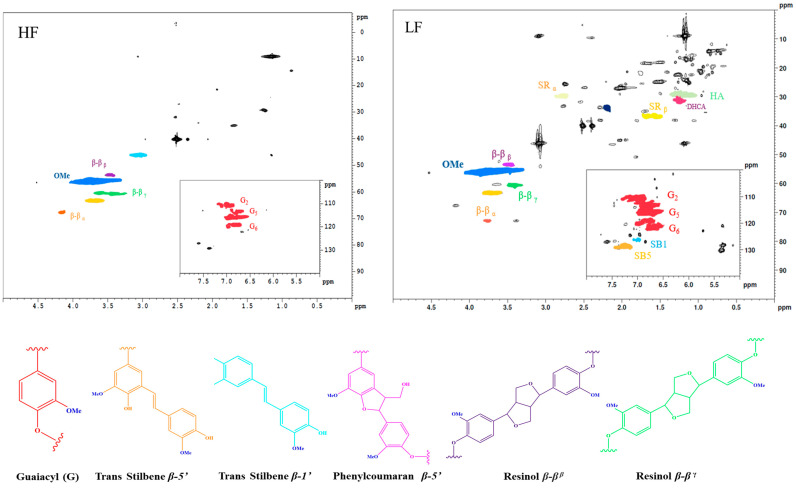
Aliphatic and aromatic HSQC NMR spectra of HF and LF.

In both the HF and LF spectra, dominant signals corresponding to the methoxy groups (Ome), *β*−*β* (resinol), and *β*-aryl-ether (Aγ) structures were observed, along with minor contributions from the *β*–5 structures and cinnamyl alcohol end groups. However, the aliphatic oxygenated side-chain region, which includes *β*-aryl-ether (A*β* and A*α*), phenylcoumaran (B*β* and B*α*), resinol (C*α*), and cinnamyl alcohol (I*γ*) end groups, exhibited lower signal intensities in the HF than in the LF. The strong resonance in the 50–86 ppm region is attributed to carbohydrate structures, indicating that the HF is rich in carbohydrates [38]. Although, the reduction in signal intensity suggests the limited solubility of HF, as evidenced by the weaker signals in the HSQC spectra (Figure 4). The decreased or absent signals for methoxyl groups, *β*−*β* (resinol), β-aryl-ether (A*γ*), and other side-chain structures in the HF indicate that the carbohydrate content significantly influences lignin solubilization. These findings align with earlier observations from ATR-FTIR and HP-SEC analyses, which highlighted the role of carbohydrates in lignin solubility and molecular weight distribution. For instance, lignin with fewer impurities typically exhibits a narrower molecular weight distribution, ranging from 1940 to 5260 g/mol [41]. In higher-molecular-weight lignins, xylose is primarily involved in benzyl ether linkages, whereas mannose participates in phenyl glycoside linkages, both of which are associated with hemicellulose [42]. Consequently, insoluble lignin fragments, which may contain carbohydrate linkages, were not detected using 2D HSQC NMR. Therefore, complementary techniques such as Py-GC/MS are necessary to fully characterize these insoluble components and their structural changes.

In the aromatic region, the HSQC spectra of the HF and LF revealed prominent cross-signals corresponding to guaiacyl (G) units (G 2,5,6), consistent with the predominance of G-rings in softwood lignin (pine) [23,43,44]. A weak signal for syringyl (S) units (S 2,6) was also observed, although no distinct S-ring peaks were detected, as expected for softwood lignin [45]. The reduced intensity of the guaiacyl unit peaks (G2, G2′, G5 + G6, and G5′) in the HF suggests the partial solubilization of the aromatic structures in DMSO, highlighting the potential influence of carbohydrates on solubility [46]. Previous studies have demonstrated that lignin is extensively linked to polysaccharides, forming lignin–carbohydrate complexes (LCCs) that may exist as large macromolecules within the cell wall matrix [19,42,47,48]. The extraction and analysis of LCCs often require ball milling to degrade the cell wall and release lignin and associated carbohydrates [49]. In this study, phenylglycoside bonds, which are preferentially solubilized by ethanol, were implicated in the formation of various phenylglycoside structures, further emphasizing the role of carbohydrates in lignin solubility [50]. Although 2D HSQC NMR is a powerful tool for elucidating lignin structures, it is limited by its inability to detect insoluble fragments. This aligns with previous reports on the challenges of solubilizing high-molecular-weight lignin fractions in organic solvents.

### 2.5. ^31^P NMR of Different Lignin Fractions

**Figure 5:**
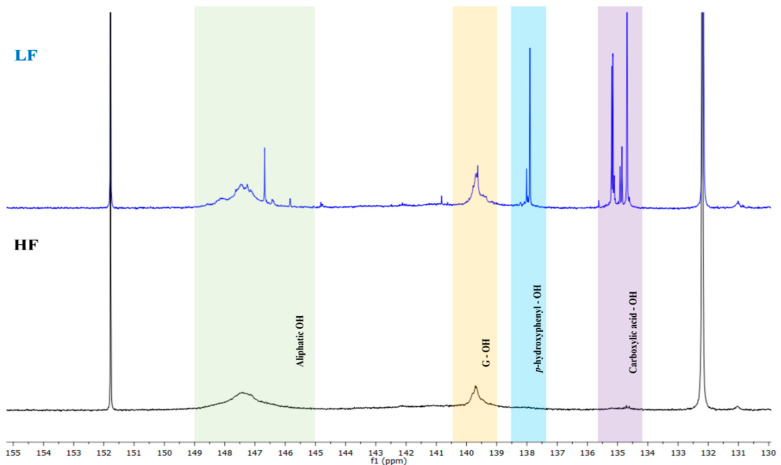
Comparison of aliphatic OH, guaiacyl OH, p-hydroxyphenyl OH, and carboxylic acid OH groups in ^31^P NMR spectra of HF and LF.

The contents of aliphatic hydroxyl groups, phenolic hydroxyl groups, 5-substituted structures, and carboxyl groups in the HF and LF were quantified using ^31^P NMR spectroscopy. The analysis revealed distinct trends in the distribution of these functional groups, providing insights into the structural changes induced by membrane fractionation. A key observation was the increase in the total hydroxyl group content as the molecular weight decreased, which was consistent with previous studies [24,29]. This trend was evident in our results, as the total hydroxyl (−OH) content of the HF (4.43 mg/g) was slightly lower than that of the LF (4.54 mg/g), with a corresponding increase in carboxylic acid content in the LF, as shown in Table 2, indicating that fractionation and the associated reduction in molecular weight led to the formation of the carboxyl groups, as shown in Figure 5. This increase in carboxylic groups is likely attributable to the cleavage of aliphatic and phenolic hydroxyl groups during fractionation, which are subsequently oxidized to carboxylic acids [25,29].

The observed decrease in the total phenolic hydroxyl content and increase in overall −OH amount after fractionation can be explained by the preferential cleavage of aliphatic hydroxyl groups and phenolic hydroxyl groups. This phenomenon aligns with the well-documented behavior of lignin during chemical and physical pretreatments, where aliphatic −OH groups are more susceptible to cleavage and conversion into carboxylic acids. Furthermore, the higher aliphatic −OH content in the HF is consistent with the presence of covalently bonded lignin structures and the abundance of high-molecular-weight polymers, which typically contain a greater proportion of hydroxyl groups. In contrast, the LF exhibited a higher concentration of low-molecular-weight compounds, likely due to lignin degradation during physical and chemical pretreatments. For instance, organosolv treatment of technical lignin (TL) has been shown to cleave *β-O-4* linkages and ester bonds, resulting in smaller lignin fragments [25]. Thus, the fractionation process explains the decreased in phenolic and increased in carboxylic hydroxyl content in LF, along with the increased presence of low-molecular-weight compounds.

### 2.6. Different Sugars Concentration in Lignins

**Figure 6:**
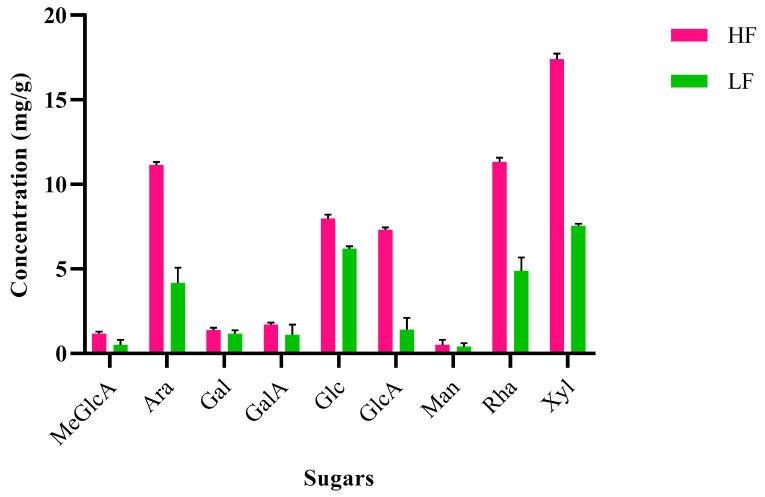
Composition of different sugars found in HF and LF [glucose (Glc), xylose (Xyl), mannose (Man), arabinose (Ara), galactose (Gal), rhamnose (Rha), glucuronicacid (GlcA), 4-O-methyl-D-glucuronic acid (MeGlcA), and galacturonic acid (GalA)].

Nine different monosaccharides, namely, glucose, xylose, mannose, arabinose, galactose, rhamnose, glucuronic acid, 4-O-methyl-D-glucuronic acid, and galacturonic acid were identified and compared between the HF and LF (Figure 6). The higher sugar content of 59.92 ± 2.12 mg/g, approximately 6% in the HF, indicates stronger LCCs, where carbohydrates such as arabinose, galactose, and uronic acids (e.g., glucuronic acid) form covalent bonds (ether/ester linkages) with lignin, as described by many authors previously [50,51]. These bonds create a rigid matrix that resists enzymatic or catalytic cleavage, reducing monomer yields by up to 20–30% compared to pure lignin. The lower sugar content of 27.37 ± 1.13 mg/g in the LF lignin implies fewer LCCs, making lignin more accessible to depolymerization agents. This improves the yields of phenolic monomers (e.g., syringol and vanillin) by 15–25% compared to the HF lignin. However, monosaccharides in lignin enhance the functionality of materials and complicate biochemical conversion. Therefore, tailoring strategies for the MW and sugar profiles of lignin is critical for high-value applications, for example, encapsulation and filtration membranes [52,53]. The structural robustness of the HF lignin suits niche material applications, whereas the processability of the LF lignin aligns with bulk biorefinery requirements. Advances in separation technologies will unlock the full potential of the lignin, as demonstrated in this study. For example, arabinose and galactose form branched structures that enhance the mechanical strength of lignin, making the HF lignin suitable for use in bio-composites [51,54]. However, rhamnose and glucuronic acid contribute to cross-linking via carboxyl and hydroxyl groups, thereby improving the thermal stability of adhesives [20]. Hence, this integrated ionic liquid pretreatment and membrane filtration method is an interesting approach for separating carbohydrate-rich and lignin-rich fractions of high-value materials, while minimizing sugar interference.

### 2.7. Py-GC/MS of HF and LF

A total of twenty-nine known compounds and six unknown compounds were detected in the LF fraction. The major peaks corresponding to guaiacol, 4-vinylguaiacol, methyl guaiacol, eugenol, cis-isoeugenol, hexadecenoic acid, and cis-13-octadecenoic acid are listed in Table 3. A similar distribution of twenty-nine known and five unknown compounds was observed in the HF fraction, as shown in Table 4. The LF and HF spectra are shown in Supplementary Information (Figures S1 and S2).

Ionic liquid pretreatment following the lignin fractionation of PWB has been suggested to disrupt interunit linkages and cleave branched chains, thereby generating aromatic compounds and organic acids [55]. These findings align with those of previous studies that utilized GPC, ATR-FTIR, 2D HSQC spectroscopy, and ^31^P NMR spectroscopy [24,26,56,57]. Notably, the increase in the peak area corresponding to organic acids was significantly greater in the LF than in the HF, likely due to the cleavage of aliphatic side chains and phenolic hydroxyl groups, resulting in the formation of carboxylic acids. This observation corroborates earlier findings that fractionation facilitates side-chain excision rather than disruption of inter-lignin bonds.

The substantial presence of low-molecular-weight compounds in the LF results from the structural breakdown of lignin during chemical and physical pretreatment. Organosolv treatment, for instance, effectively cleaves *β-O-4* linkages and ester bonds, leading to the enhanced depolymerization of lignin [58]. High-molar-mass lignins with broader dispersity are particularly attractive for material applications, such as thermoplastic and thermosetting polymer blends. However, the HF yield remains a limiting factor for lignin valorization. The application potential of lignin-based materials can be significantly expanded by fractionating the lignin post-pretreatment, as proposed in this study. Additionally, fractionation at various pretreatment temperatures facilitates the incorporation of “hydroxyalkylated” groups due to carbohydrate linkages at elevated temperatures, thereby altering the physicochemical properties of lignin [23]. Furthermore, the HF displays significant resistance to organic solvents, whereas the LF solubilizes efficiently, making it a promising candidate for various high-value applications [25]. However, this solvent resistance also presents challenges for the further characterization of its monomeric and oligomeric constituents. Identifying suitable solvents for HF dissolution would facilitate more comprehensive structural and compositional analyses, as highlighted in previous lignin solubility studies [59]. These findings suggest that future research should focus on optimizing the fractionation methods to tailor the thermal and solubility properties of lignin for targeted applications in advanced materials.

## 3. Materials and Methods

### 3.1. Reagents and Biomass Preparation

Triethylamine (>99%) and DMSO-d6 (99.9% D) were bought from Sigma-Aldrich (Steinheim, Germany), and methanesulfonic acid (70% aq.) was bought from Carl Roth (Karlsruhe, Germany). Domestically produced raw scots pine (*Pinus sylvestris*), a softwood, was used for this study. The biomass moisture content was analyzed using a Kern MLS-50-3D moisture analyzer (Kern & Sohn GmbH, Balingen, Germany), whereas fiber content was determined using an Ankom 2000 fiber analyzer (ANKOM Technology, Fairport, NY, USA). The collected pinewood was debarked, air-dried, ground to 1–2 mm, maintained at <10% moisture, and stored in an air-tight container while triethylamine was distilled prior to use.

### 3.2. Protic Ionic Liquid (PIL) Preparation and Its Characterization

Triethylammonium methanesulfonate ([N222H][OMS]) was synthesized according to the established protocols [60]. [N222H][OMS] was synthesized by dropwise neutralization of 1.1 equivalents of aqueous triethylamine solution with 1.0 equivalent of aqueous methanesulfonic acid at 0 °C. The resulting solution was stirred for 3 h, the excess of triethylamine and solvent were removed by rotary evaporation, and the resulting residue was dried under vacuum at 70 °C for 2 d, yielding the PIL in quantitative amounts as a colorless crystalline solid. 1H-NMR (400 MHz, DMSO-d6): δ 9.30 (s, 1 H, NH), 3.41–2.95 (m, 6 H, CH2), 1.30 (t, J = 7.3 Hz, 9 H, -CH3); 13C-NMR (101 MHz, DMSO-d6) δ 45.71 (CH2), 38.89(SO3CH3), 8.55 (CH2CH3).

### 3.3. Pine Wood Pretreatment

Dried ground pinewood was combined with [N222H][OMS] at a 1:3 (*w*/*w*) ratio in a 100 mL pressure tube. The mixture was vortexed and heated at 180 °C for 90 min. Following the reaction, ethanol was added to facilitate the separation of the PIL-lignin mixture from the cellulose-rich pulp, which was then isolated by centrifugation for 10 min at 4000 rpm (repeated three times).

### 3.4. Lignin Fractionation

The ethanol-soluble lignin-PIL mixture underwent vacuum filtration through a 0.8 µm nylon membrane, yielding high-molecular-weight lignin fractions (HF, retentate) and low-molecular-weight lignin fractions (LF, permeate). PIL removal was achieved by antisolvent precipitation with deionized water (1:5 *v*/*v*), followed by centrifugation (4000 rpm, 10 min). The precipitated lignin fractions were washed thrice with Milli-Q water, air-dried, and desiccated for analysis. The residual ethanol was recovered using a rotary vacuum evaporator (Büchi Rotavapor R-200, Büchi, Switzerland).

### 3.5. Delignification Quantification

**Figure 7:**
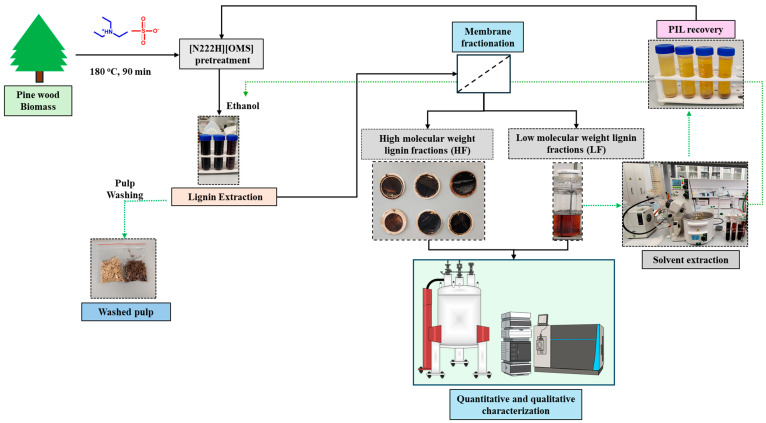
Overview of lignin extraction and its fractionation from PWB biomass using [N222H][OMS].

Post-pretreatment, ethanol was used to facilitate the separation of the PIL-lignin mixture from cellulose pulp via centrifugation. The ethanol-soluble fraction was vacuum-filtered (0.8 µm nylon) to isolate high-molecular-weight (HF, retentate) and low-molecular-weight (LF, permeate) lignin fractions (Figure 7). PIL was removed from both fractions by antisolvent precipitation using water, followed by centrifugation, washing, air-drying, and desiccation. The purified HF and LF lignins were quantified gravimetrically. The delignification efficiency (%) and lignins yield were calculated using established formulas based on the initial and posttreatment lignin content, as described in our previous work [61].

### 3.6. Lignin Characterization

#### 3.6.1. Molecular Weight Analysis

A Gel permeation chromatography (GPC) device (Shimadzu Prominence-i LC-2030C 3D Plus, Shimadzu Corporation, Kyoto, Japan), equipped with MCX columns (1000 Å, 100,000 Å), a UV detector (280 nm), and LabSolutions GPC Postrun analysis software (Version 5.71 SP1, Shimadzu, Kyoto, Japan), was used to determine the molecular weight of lignin. Samples, prepared at 5 mg/mL in 0.1 M NaOH, were eluted isocratically (0.5 mL/min) and calibrated against polystyrene sulfonate standards (1.1–100 kDa) [61].

#### 3.6.2. Nuclear Magnetic Resonance (2D HSQC and ^31^P NMR) Spectroscopy

For 2D HSQC, 80 mg of lignin was dissolved in DMSO-d6 and analyzed on a Bruker 500 MHz spectrometer (Bruker Ltd., Fällanden, Switzerland) (hsqcetgpsi pulse sequence, 256 increments, 80 scans). The spectral width was set to 8012 Hz (from −3.3 to16 ppm) in the F2 axis and 20,750 Hz (−7.5 to 157.5 ppm) along the F1 axis. For the quantitative ^31^P NMR (Bruker 202.46 MHz), 20.0 mg of freeze-dried powders were dissolved in pyridine/CDCl3 and quantified hydroxyl groups after phosphorylation with 2-chloro-4,4,5,5-tetramethyl-1,3,2-dioxaphospholane for 20 min. Chromium(III) acetylacetonate was used as the relaxation reagent, and endo-N-hydroxy-5-norbornene-2,3-dicarboximide was used as the internal standard [29].

#### 3.6.3. ATR-FTIR (Attenuated Total Reflectance/Fourier Transform Infrared) Analysis of Lignin Fractions

The functional groups were analyzed using a Bruker Alpha (Fourier transform infrared) FTIR spectrometer equipped with an Attenuated total reflectance (ATR) accessory (Bruker, Billerica, MA, USA). Spectra (32 scans, 4000–600 cm^−1^, 4 cm^−1^ resolution) were used to identify the bond vibration characteristics of the lignin substructures.

#### 3.6.4. Carbohydrate Quantification of Lignin Fractions

The sugar content was quantified via acid methanolysis (2 M HCl/MeOH, 105 °C, 4 h) with 10 mg (± 0.01 mg) of the freeze-dried sample. After neutralization with pyridine, the sample was derivatized overnight using HMDS/THMS, followed by the addition of 1.0 mL of an internal standard (0.1 mg/mL resorcinol in methanol). The silylated analytes were separated using an HP-1 GC column (Shimadzu GC-2010 AF) with hydrogen carrier gas (1 mL/min) and FID detection (Shimadzu, Kyoto, Japan).

#### 3.6.5. Pyrolysis-GC/MS

For the pyrolysis-GC/MS analysis, approximately 100 μg of freeze-dried sample was placed on a platinum filament and pyrolyzed at 650 °C (Pyrola 2000), coupled to an Agilent 7890 B GC/5977B MS (ZB-35 column) (Agilent Technologies, Santa Clara, CA, USA). The resulting pyrolysis products were analyzed, with the oven temperature ramping from 50 °C (held for 0.5 min) to 320 °C (8 °C/min), with EI-MS scanning at *m*/*z* 35–700.

## 4. Conclusions

This study demonstrated a biorefinery strategy utilizing the protic ionic liquid (PIL) [N222H][OMS] and membrane to effectively overcome high carbohydrate contamination in lignin valorization. We presented an integrated lignin extraction and fractionation method with low carbohydrate contamination, confirmed by advanced analysis (NMR, Py-GC/MS, GPC). This demonstrates the PIL’s dual efficacy and its selectivity while simultaneously fractionating the lignin using the membrane into two distinct fractions, the HF and LF. The PIL thus serves as both an efficient extraction medium and in situ modification agent, enabling the production of structurally defined lignins suitable for high-value applications. The fractions derived in this study offer a promising route toward environmentally benign, bio-based products that do not require extensive chemical modifications. Furthermore, lignin fractions with variable molar mass distributions (HF) can be used in diverse applications such as dispersants, emulsifiers, colloids, coatings, paints, and adhesives. When infused with active ingredients, fractionated lignin (LF) can be engineered into self-healing materials and stimuli-responsive controlled-release systems for use in agricultural products (e.g., biocides and plant growth regulators), cosmetics, nanomedicine, and related fields. Given the advantages of fractionated lignins, integrated PIL pretreatment with lignin fractionation is a promising approach for advanced applications.

## Figures and Tables

**Table 1:** Molecular weights and PDI of HF and LF.

Lignin	Mn	Mw	PDI
HF	1452	9535	6.56
LF	841	3342	3.97

M_n_—number average molecular weight, M_w_—weight average molecular weight, PDI—polydispersity index (M_w_/M_n_).

**Table 2:** ^31^P NMR quantification of HF and LF.

	Aliphatic-OH ^a^	Phenolic-OH from Lignin			Total Phenolic-OH	Carboxylic Acid OH ^e^	Total OH
		C_5_-Substituted OH ^b^	Guaiacyl-OH ^c^	*p*-Hydroxyphenyl-OH ^d^			
HF	1.97	1.09	0.93	0.21	2.23	0.23	4.43
LF	1.69	0.64	0.89	0.42	1.95	0.90	4.54

^a^ integral of 150–145 ppm; ^b^ integral of 145–140.8 ppm; ^c^ integral of 140.6–138.8 ppm; ^d^ integral of 138.6–137.1 ppm; ^e^ integral of 136–133.6 ppm.

**Table 3:** Peak assignment and their proportions from the Py-GC/MS chromatogram of LF.

Retention Time (min)	Compound	Chemical Formula	Py–GC/MS Peak Area (%)
**Phenol-type (H)**			
6.58	Phenol	C_6_H_6_O	0.45
7.91	p-Creosol	CH_3_C_6_H_4_OH	0.57
**Guaiacyl-type (G)**			
8.52	Guaiacol	C_7_H_8_O_2_	5.79
12.48	4-Vinylguaiacol	C_9_H_10_O	10.32
11.95	4-Ethylguaiacol	C_9_H_12_O_2_	3.20
13.53	4-Propylguaiacol	C_10_H_14_O_2_	0.86
9.68	2,4-Dimethylphenol	C_8_H_10_O	0.93
10.46	Methyl guaiacol	C_8_H_10_O_2_	19.36
13.46	Eugenol	C_10_H_12_O_2_	2.21
14.92	Cis-Isoeugenol	C_10_H_12_O_2_	9.54
15.23	Homo vanillin	C_9_H_10_O_3_	0.85
15.54	Acetovanillone	C_10_H_10_O_4_	2.91
16.12	Guaiacyl acetone	C_10_H_12_O_3_	1.83
16.89	Guaiacyl propenol	G-C_3_H_6_O	1.28
16.93	1-guaiacyl-2-propen-1-ol	G-C_3_H_6_O	1.18
17.98	Dihydro coniferyl alcohol	C_10_H_14_O_3_	1.66
**Catechol-type (Ca)**			
10.92	Catechol	C_6_H_6_O_2_	0.84
11.88	4-Methylcathecol	C_7_H_8_O_2_	1.93
**Other aromatic compounds**			
12.53	Veratrole	C_6_H_4_(OCH_3_)_2_	0.46
23.18	4-(1-propenyl) veratrole	C_6_H_4_(OCH_3_)_2_-CH=CH-CH_3_	0.3
**Fatty acids**			
19.78	Hexadecenoic acid	C_16_H_30_O_2_	0.39
23.93	cis-13-octadecenoic acid	C_18_H_34_O_2_	3.97
**Other identified compounds**			
1.12	Carbon dioxide	CO_2_	11.85
2.38	Triethylamine	N(CH_2_CH_3_)_3_	2.07
3.06	Methylbenzene	C_6_H_5_CH_3_	1.53
2.08	Benzene	C_6_H_6_	0.29
8.92	2,3-Dimethylanisole	C_9_H_12_O	0.49
1.82	2-Methyl-furan	C_5_H_6_O	1.5
**Unidentified compounds**			
1.36	Unknown	-	3.05
2.24	Unknown	-	4.54
19.22	Unknown	-	0.79
19.57	Unknown	-	0.38
23.35	Unknown	-	0.72
25.92	Unknown	-	1.04

**Table 4:** Peak assignment and their proportions from the Py-GC/MS chromatogram of HF.

Retention Time (min)	Compound	Chemical Formula	Py–GC/MS Peak Area (%)
**Phenol-type (H)**			
6.58	Phenol	C_6_H_6_O	0.41
7.92	p-Creosol	CH_3_C_6_H_4_OH	0.64
**Guaiacyl-type (G)**			
8.52	Guaiacol	C_7_H_8_O_2_	4.23
10.43	3-Methylguaiacol	C_8_H_10_O_2_	0.31
12.62	4-Vinylguaiacol	C_9_H_10_O	7.88
11.92	4-Ethylguaiacol	C_9_H_12_O_2_	2.33
13.43	4-Propylguaiacol	C_10_H_14_O_2_	0.66
9.61	2,4-Dimethylphenol	C_8_H_10_O	1.01
10.43	Methyl guaiacol	C_8_H_10_O_2_	16.31
13.24	Eugenol	C_10_H_12_O_2_	2.23
14.76	Trans-Isoeugenol	C_10_H_12_O_2_	9.29
15.13	Hom vanillin	C_9_H_10_O_3_	1.29
15.52	Acetovanillone	C_10_H_10_O_4_	0.97
16.13	Guaiacyl acetone	C_10_H_12_O_3_	1.54
16.83	Guaiacyl propenol	Guaiacyl propenol	1.53
16.92	1-guaiacyl-2-propen-1-ol	G-C_3_H_6_O	1.06
17.92	Dihydro coniferyl alcohol	C_10_H_14_O_3_	1.01
**Other aromatic compounds**			
13.89	Veratrole	C_6_H_4_(OCH_3_)_2_	0.33
13.97	4-(1-propenyl) veratrole	C_6_H_4_(OCH_3_)_2_-CH=CH-CH_3_	0.23
**Carbohydrate units**			
15.71	1,6-anhydro-β-d-glucopyranose	C_6_H_10_O_5_	2.42
**Other identified compounds**			
1.15	Carbon dioxide	CO_2_	17.37
2.23	Triethylamine	N(CH_2_CH_3_)_3_	10.28
3.06	benzene, methyl- (cas)	C_6_H_5_CH_3_	1.18
3.02	1-methoxy-3-methylbenzene	C_6_H_5_CH_3_	0.16
8.88	2,3-dimethylanisole	C_9_H_12_O	0.33
13.25	2,6-dimethyl-3(2h)-benzofuranone,	C_10_H_10_O_2_	0.14
28.09	Bis(ethylhexyl)phthalate	C_6_H_4_(COOC_8_H_17_)_2_	6.69
**Unidentified compounds**			
1.35	Unknown	-	4.47
15.27	Unknown	-	0.38
15.38	Unknown	-	0.58
15.74	Unknown	-	0.57
16.97	Unknown	-	1.29

## Data Availability

The original contributions presented in this study are included in the article/Supplementary Material. Further inquiries can be directed to the corresponding authors.

## Supplementary Materials

The following supporting information can be downloaded at: https://www.mdpi.com/article/10.3390/molecules30122630/s1, Figure S1: Py-GC/MS spectra of HF, Figure S2: Py-GC/MS Spectra of LF, Table S1: HSQC NMR peak assignments. References [23,25,29,43,45,62,63,64,65,66] are used in the Supplementary Information Table S1.
