# Supplementary material for: Tailored Lignin Fractions via Ionic Liquid Pretreatment for Sustainable Polymer Systems
Source: Molecules. 2025 Jun 17;30(12):2630. doi: 10.3390/molecules30122630 (PMC12195699; doi:10.3390/molecules30122630)
Supplement: Supplementary file 1 [file molecules-30-02630-s001.zip › molecules-3660154-supplementary.pdf]

Supplementary Information for

Tailored Lignin Fractions Via Ionic Liquid Pretreatment for Sustainable Polymer Systems

Sharib Khan [1,*], Daniel Rauber [2], Udayakumar Veerabagu [1], Ruijie Wu [3], Christopher W. M. Kay [2, 4], Chunlin Xu [3], Sabarathinam Shanmugam [1,*], Timo Kikas [1,*]

[1] Biosystems Engineering, Institute of Forestry and Engineering, Estonian University of Life Sciences, Kreutzwaldi 56, 51006 Tartu, Estonia

[2] Department of Chemistry, Saarland University, Campus B2.2, 66123 Saarbrücken, Germany

[3] Laboratory of Natural Materials Technology, Åbo Akademi University, Henrikinkatu 2, Turku, FI-20500 Finland

[4] London Centre for Nanotechnology, University College London, 17-19 Gordon Street, London WC1H 0AH, UK

* Correspondence: sharib.khan@emu.ee, sabarathinam.shanmugam@emu.ee , timo.kikas@emu.ee

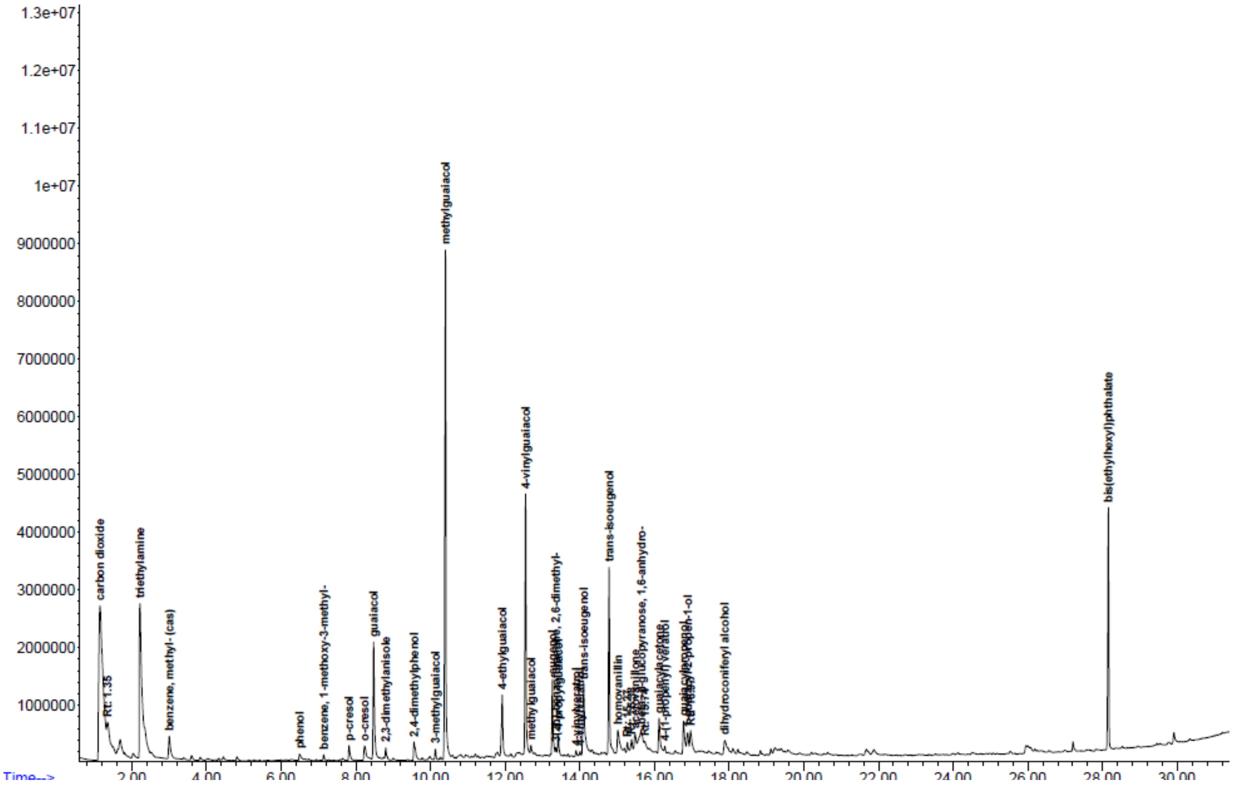

Figure S1: *Py-GC/MS Spectra of HF*

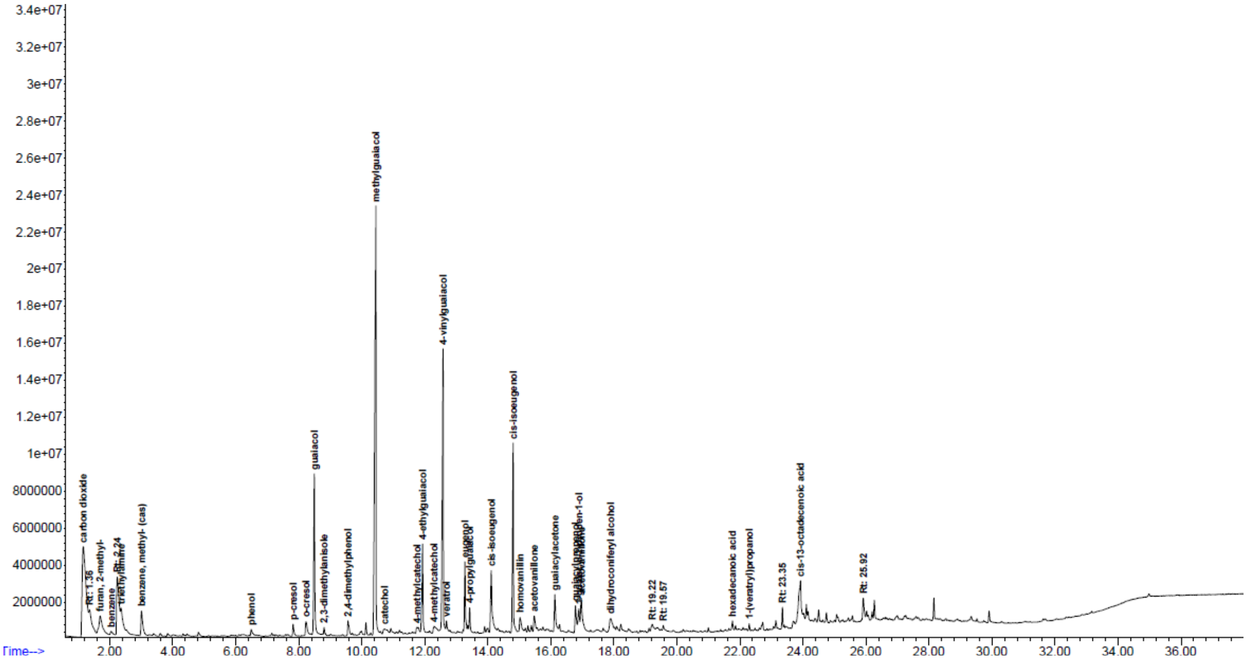

Figure S2: *Py-GC/MS Spectra of LF*

Table S1: *HSQC NMR peak assignments*

Name	Peak assignments
pinoresinol (β-β')	δ_C/δ_H β: 54.21/3.08, γ: 71.45/4.23 and 3.85
epiresinol (β-β')	δ_C/δ_H γ: 70.8/3.75 and 4.12
dihydro cinnamyl alcohol	δ_C/δ_H α: 31.97/2.55, γ: 60.73/3.43
aryl glycerol	δ_C/δ_H γ: 63.4/3.47
guaiacyl unit	δ_C/δ_H G2: 110.9/6.91, G5: 114.9/6.86<br>G6: 119.2/6.77
Methoxyls (MeO)	δ_C/δ_H 55.5/3.66
phenylcoumaran (β-5)	δ_C/δ_H 86.8/5.49 (α), 54.5/3.47 (β), 63.9/3.73/3.62 (γ)
resinol (β-β)	δ_C/δ_H 85.1/4.63 (α), 54.2/3.07 (β), 70.9/4.16/3.66 (γ)
trans-stilbene (SB1) and SB5	δ_C/δ_H 125.0/7.01 (α), δC/δH 128.0/7.12 (α), 120.0/7.22 (β)
Carbohydrate, β-D-xylopyranoside	X1: 102.3/4.30, X2: 73.2/3.08<br>X3: 74.6/3.29, X4: 75.9/3.54<br>X5: 63.8/3.92 and 3.23
